# Association between ADAMTS13 deficiency and cardiovascular events in chronic hemodialysis patients

**DOI:** 10.1038/s41598-021-02264-5

**Published:** 2021-11-24

**Authors:** Shih-Yuan Hung, Tsun-Mei Lin, Hung-Hsiang Liou, Ching-Yang Chen, Wei-Ting Liao, Hsi-Hao Wang, Li-Chun Ho, Ching-Fang Wu, Yi-Che Lee, Min-Yu Chang

**Affiliations:** 1 School of Medicine for International Students, College of Medicine, I-Shou University, Kaohsiung, Taiwan; 2 Division of Nephrology, Department of Internal Medicine, E-DA Hospital, No. 1, Yida Road, Jiaosu Village, Yanchao District, Kaohsiung City, 82445 Taiwan; 3 Department of Medical Laboratory Science, I-Shou University, Kaohsiung, Taiwan; 4 Department of Medical Research, E-DA Hospital, Kaohsiung, Taiwan; 5 Division of Nephrology, Department of Internal Medicine, Hsin-Jen Hospital, New Taipei City, Taiwan; 6 Department of Laboratory Medicine, E-DA Hospital, Kaohsiung, Taiwan

**Keywords:** Biomarkers, Cardiology, Diseases, Medical research, Nephrology, Risk factors

## Abstract

A mild decrease of ADAMTS13 (a disintegrin and metalloprotease with thrombospodin type 1 motif 13) could attribute to stroke and coronary heart disease in general population. However, the role of ADAMTS13 in hemodialysis (HD) patients remains to be explored. This cross-sectional and observational cohort study enrolled 98 chronic HD patients and 100 normal subjects with the aims to compare the ADAMTS13 activity between chronic HD patients and normal subjects, and to discover the role of ADAMTS13 on the newly developed cardiovascular events for HD patients in a 2-year follow-up. Our HD patients had a significantly lower ADAMTS13 activity than normal subjects, 41.0 ± 22.8% versus 102.3 ± 17.7%, *p* < 0.001. ADAMTS13 activity was positively correlated with diabetes, triglyceride and hemoglobin A1c, and negatively with high-density lipoprotein cholesterol levels in HD patients. With a follow-up of 20.3 ± 7.3 months, the Cox proportional hazards model revealed that low ADAMTS13, comorbid diabetes, and coronary heart diseases have independent correlations with the development of cardiovascular events. Our study demonstrated that chronic HD patients have a markedly decreased ADAMTS13 activity than normal subjects. Although ADAMTS13 seems to correlate well with diabetes, high triglyceride and low high-density lipoprotein cholesterol levels, ADAMTS13 deficiency still carries an independent risk for cardiovascular events in chronic HD patients.

## Introduction

ADAMTS13 (a disintegrin and metalloprotease with thrombospodin type 1 motif 13), a specific von Willebrand factor (vWf)-degrading protease, was discovered by Tsai and Furlan et al. in 1996^1,2^. It belongs to a zinc-containing metalloprotease family and is responsible for cleaving vWf into less active fragments. Therefore, deficiency of ADAMTS13 causes an accumulation of ultra-large vWf multimers in the plasma through which induces pathogenic thrombosis. Both in prospective and meta-analysis studies, high vWf levels had been demonstrated to enhance the risks of coronary heart disease (CHD) and ischemic stroke^3–6^. As a consensus of the International Working Group for Thrombotic Thrombocytopenic Purpura in 2017, thrombotic thrombocytopenic purpura can be distinguished from other causes of thrombotic microangiopathies by the finding of severe ADAMTS13 deficiency to less than 10%^7^. Accordingly, severe deficiency of ADAMTS13 has been regarded as a hallmark for thrombotic thrombocytopenic purpura^8^.

It deserves clinical interests while the ADAMTS13 activity is only mildly decreased. In 2007 and 2008 Crawley et al. first conducted 2 cross-sectional studies focusing on ADAMTS13 and myocardial infarction (MI)^9,10^. In the study including 466 MI cases and 484 age- and sex-matched controls, they found that low ADAMTS13 levels were independently correlated with high risk of MI after adjusting vWf and multiple risk factors^10^. They concluded that the cases in the low tertile carried a significantly high risk of MI. The ADAMTS13 level for the low tertile was less than 95.8%, though the mean value for all subjects was 111.0%^10^. In 2012, two animal studies on ADAMTS13 knockout mice gave further evidence. Both showed that ADAMTS13-deficient mice have larger MI area and myocytes apoptosis compared with wild-type mice upon induction of myocardial ischemia^11,12^.

The first prospective cohort study, reported by Sonneveld et al. in 2015, enrolled 5941 individuals aged ≥ 55 years without history of stroke or transient ischemic attack and followed them up to 12 years. They found that low ADAMTS13 activity is an independent risk factor of ischemic stroke. The mean ADAMTS13 activities of the lowest quartile and the total cohort were 70.3% and 91.9%, respectively^13^. Subsequently, in 2016, these authors issued another investigation focusing on CHD. In 5688 adults aged ≥ 55 years without history of CHD, they discovered that patients in the lowest quartile were associated with an increased risk of CHD in a 12-year of follow-up, independent of vWf levels and several well-known cardiovascular risk factors^14^. Based on these results, even a mild decrease of ADAMTS13 activity could be a risk factor for ischemic stroke and CHD in general population.

Patients who undergoing hemodialysis (HD) suffered from higher prevalence of CHD and stoke^15,16^, however, the impacts of ADAMTS13 on cardiovascular diseases of HD patients are not clearly defined. By now there were found only 2 studies investigating ADAMTS13 in HD patients. But, both focused on vascular access thrombosis and the results were inconclusive. In a cross-sectional study including 195 HD patients and 80 healthy controls in 2012, Rios et al. concluded that the decrease of ADAMTS13 does not explain the occurrence of vascular access thrombosis^17^. However, another cross-sectional study performed by Elzorkany et al. in 2018, found that the patients with vascular access thrombosis had significantly lower ADAMTS13 levels than those without^18^. Despite their controversial results, both studies revealed that HD patients had significantly lower levels of ADAMTS13 compared to that in normal subjects. Therefore, a study focusing on ADAMTS13 and cardiovascular diseases in HD patients is expecting.

In the present study, we aimed to compare the ADAMTS13 activity between chronic HD patients and normal subjects, to explore the correlations between ADAMTS13 and multiple cardiovascular risk factors in HD patients, and to discover the role of ADAMTS13 on the newly developed cardiovascular events in HD patients.

## Results

**Figure 1:**
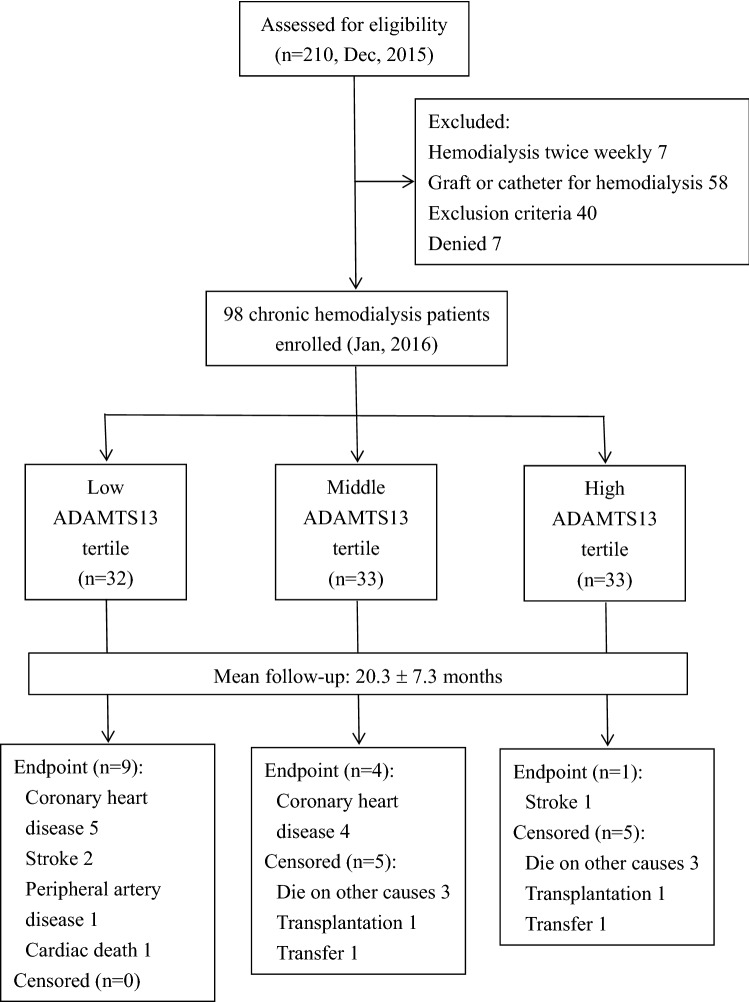
Flow diagram detailing the participants flow.

Among 210 patients under regular HD therapy at E-DA hospital, 112 were excluded based on the criteria. The details were shown in Fig. 1. Finally, 98 of them who aged 62.6 ± 11.3 years and 39 (39.8%) were males participated in this study. The underlying causes of end-stage renal disease were diabetes (43 patients, 43.9%), chronic glomerulonephritis (37 patients, 37.8%), hypertension (7 patients, 7.1%), chronic interstitial nephritis (7 patients, 7.1%), and others (4 patients, 4.1%).

### ADAMTS13 activity between HD and healthy subjects

The 100 age- and sex-matched healthy subjects had a mean age of 62.1 ± 1.6 years, and 40 (40.0%) of them were males. Our 98 HD patients demonstrated a significantly lower ADAMTS13 activity than that of healthy subjects (41.0 ± 22.8% vs. 102.3 ± 17.7%, *p* < 0.001).

### ADAMTS13 activity in HD patients

Among all the demographic and clinical data, ADAMTS13 activity was positively correlated with triglyceride (r = 0.425, *p* < 0.001), HbA1c (r = 0.376, *p* < 0.001) and the presence of DM (r_s_ = 0.258, *p* = 0.01), but negatively with HDL levels (r = − 0.229, *p* = 0.02) only. While equally divided all HD patients into 3 groups according to their ADAMTS13 activities, we found that only serum calcium (*p* = 0.02), fasting glucose (*p* = 0.02), HbA1c (*p* < 0.001) and triglyceride (*p* = 0.002) levels showed significant differences among groups (Table 1). Still, fasting glucose, HbA1c and triglyceride levels demonstrated positive correlations with the ADAMTS13 activity.

**Table 1:** Demographic and laboratory data of 98 chronic hemodialysis patients and the comparisons among three tertile groups according to ADAMTS13 activity.

n	All patients	Low tertile	Middle tertile	High tertile	*p*
	98	32	33	33	
ADAMTS13 activity (%)	40.1 ± 22.8	18.9 ± 6.9	37.2 ± 4.5^a^	66.1 ± 18.9^ab^	< 0.001
Age (years)	62.6 ± 11.3	63.7 ± 8.2	62.4 ± 13.4	61.6 ± 11.7	0.8
Gender/female: n (%)	39 (39.8)	14 (43.8)	10 (30.3)	15 (45.5)	0.4
Hemodialysis vintage (month)	48.6 ± 34.1	58.0 ± 38.5	47.5 ± 33.4	40.4 ± 28.4	0.1
vWF antigen (%)	172.9 ± 69.1	167.8 ± 63.5	176.5 ± 90.4	174.7 ± 49.3	0.9
vWF activity (%)	153.7 ± 66.8	163.5 ± 78.3	138.9 ± 53.4	158.9 ± 66.0	0.4
White blood cell count (10^3^/ml)	6.27 ± 1.9	5.9 ± 1.9	6.3 ± 1.8	6.6 ± 2.0	0.3
Hemoglobin (g/dl)	10.7 ± 1.0	10.6 ± 1.1	10.6 ± 1.0	10.9 ± 0.9	0.3
Platelet (10^3^/ml)	181.1 ± 41.5	167.6 ± 41.2	190.5 ± 50.6	184.8 ± 30.3	0.3
Albumin (g/dl)	4.0 ± 0.2	4.0 ± 0.2	4.0 ± 0.2	3.9 ± 0.2	0.7
Blood urea nitrogen (mg/dl)	56.7 ± 12.8	55.6 ± 13.5	54.2 ± 12.5	58.4 ± 12.2	0.4
Creatinine (mg/dl)	10.0 ± 2.2	10.0 ± 1.9	10.2 ± 2.3	9.8 ± 2.3	0.8
Calcium (mg/dl),	9.6 ± 0.8	9.8 ± 0.8	9.3 ± 0.7^a^	9.5 ± 0.8	0.02
Phosphate (mg/dl)	5.1 ± 1.2	5.0 ± 1.2	5.2 ± 1.3	5.1 ± 1.1	0.8
Uric acid (mg/dl)	7.2 ± 1.3	7.0 ± 1.4	7.2 ± 1.4	7.6 ± 1.2	0.2
Glucose, fasting (g/dl)	114.3 ± 48.8	98.8 ± 28.9	111.4 ± 43.5	132.2 ± 63.8^a^	0.02
HbA1c (%)	6.2 ± 1.4	5.7 ± 1.0	6.0 ± 1.2	7.0 ± 1.6^ab^	< 0.001
Cholesterol, total (mg/dl)	161.6 ± 34.1	163.9 ± 33.5	154.7 ± 35.3	166.2 ± 33.4	0.3
Triglyceride (mg/dl)	149.0 ± 80.5	115.1 ± 57.5	147.3 ± 83.5	183.4 ± 84.1^a^	0.002
HDL (mg/dl)	39.2 ± 13.0	41.9 ± 14.4	38.6 ± 12.4	37.0 ± 11.9	0.3
LDL (mg/dl)	73.7 ± 24.4	76.3 ± 25.9	71.4 ± 24.0	73.4 ± 23.7	0.7
Ferritin (ug/l)	311.3 ± 402.4	272.5 ± 205.8	294.2 ± 274.2	365.6 ± 605.7	0.6
Homocysteine (umole/l)	23.2 ± 5.5	24.9 ± 5.8	22.9 ± 4.4	21.9 ± 5.9	0.07
hsCRP (mg/L)	8.5 ± 15.6	9.1 ± 17.2	6.4 ± 9.4	10.1 ± 18.9	0.6
Medications: n (%)					
Statin/ezetimibe	33 (33.7)	12 (37.5)	10 (30.3)	11 (33.3)	0.8
Aspirin	37 (37.8)	13 (40.6)	10 (30.3)	14 (42.4)	0.5
Clopidogrel	23 (23.5)	8 (25.0)	6 (18.2)	9 (27.3)	0.7
Cilostazol	5 (5.1)	1 (3.1)	2 (6.1)	2 (6.1)	0.8
Unfractionated heparin	60 (61.2)	19 (59.4)	22 (66.7)	19 (57.6)	0.7
LMWH	28 (28.6)	12 (37.5)	6 (18.2)	10 (30.3)	0.2
ACEI/ARB	35 (35.7)	14 (43.8)	7 (21.2)	14 (43.8)	0.1
Beta blocker	29 (29.6)	11 (34.4)	8 (24.2)	10 (30.3)	0.7
CCB	50 (51.0)	19 (59.4)	15 (45.5)	16 (48.5)	0.5
Comorbidity: n (%)					
DM	60 (61.2)	15 (46.9)	20 (60.6)	25 (75.8)	0.06
Hypertension	87 (88.8)	27 (84.4)	29 (87.9)	31 (93.9)	0.5
CHD	32 (32.7)	14 (43.8)	6 (18.2)	12 (36.4)	0.08
CVD	7 (7.1)	1 (3.1)	1 (3.0)	5 (15.2)	0.09
PAD	6 (6.1)	1 (3.1)	2 (6.1)	3 (9.1)	0.6

vWF = von Willebrand factor, HbA1c = hemoglobin A1c, HDL = high-density lipoprotein cholesterol, LDL = low-density lipoprotein cholesterol, hsCRP = high sensitivity C-reactive protein, LMWH = low molecular weight heparin, ACEI/ARB = angiotensin-converting enzyme inhibitor/angiotensin II receptor blocker, CCB = calcium-channel blocker, DM = diabetes mellitus, CHD = coronary heart disease, CVD = cerebral vascular disease, PAD = peripheral artery disease.

^a^*p* < 0.05 versus low tertile, ^b^*p* < 0.05 versus middle tertile (Post Hoc analysis using Scheffe test).

### Two-year follow-up for HD patients

With a mean follow-up period of 20.3 ± 7.3 months, 14 patients reached the endpoints including 9 CHD, 3 CVD, 1 PAD and 1 cardiac death. Additionally, another 10 patients were censored due to 6 died on other causes (5 septic shock and 1 intracranial hemorrhage), 2 were transferred to other hospital and 2 received kidney transplantation (Fig. 1). The Cox proportional hazards analyses were then performed. Univariate analyses showed that ADAMTS13 activity, current uses of aspirin and clopidogrel, and the comorbid DM and CHD have a p value less than 0.1, and thus were selected into the multivariate analyses. By using the method of “forward stepwise selection”, the Cox proportional hazards model showed that only ADAMTS13 activity (Hazard ratio 0.94, 95% CI 0.90–0.98, *p* = 0.003), the comorbid diseases: DM (Hazard ratio 5.60, 95% CI 1.15–27.53, *p* = 0.03) and CHD (Hazard ratio: 4.27, 95% CI 1.29–14.09, *p* = 0.02) have significant correlations with the occurrence of endpoints (Table 2).

**Table 2:** Univariate and multivariate Cox proportional hazards models for the occurrence of new cardiovascular events in the 2-year follow-up period for 98 chronic hemodialysis patients.

Variables	Univariate		Multivariate^a^	
	Hazard ratio (95% CI)	*p*	Hazard ratio (95% CI)	*p*
ADAMTS13 activity (%)	0.95 (0.92–0.99)	0.01	0.94 (0.90–0.98)	0.003
Age (years)	0.99 (0.95–1.04)	0.7		
Gender: male versus female	1.68 (0.53–5.34)	0.4		
Hemodialysis vintage (month)	1.00 (0.99–1.02)	0.8		
vWF antigen (%)	1.00 (0.99–1.01)	0.7		
vWF activity (%)	1.00 (0.99–1.01)	0.9		
White blood cell count (10^3^/ml)	1.07 (0.82–1.39)	0.6		
Hemoglobin (g/dl)	1.17 (0.71–1.93)	0.5		
Platelet (10^3^/ml)	1.00 (0.99–1.01)	0.9		
Albumin (g/dl)	2.07 (0.24–18.02)	0.5		
Blood urea nitrogen (mg/dl)	1.01 (0.97–1.05)	0.7		
Creatinine (mg/dl)	0.93 (0.73–1.12)	0.6		
Calcium (mg/dl),	1.30 (0.68–2.49)	0.4		
Phosphate (mg/dl)	1.24 (0.79–1.95)	0.3		
Uric acid (mg/dl)	0.80 (0.53–1.21)	0.3		
Glucose, fasting (g/dl)	1.00 (0.99–1.01)	0.7		
HbA1c (%)	1.09 (0.75–1.59)	0.6		
Cholesterol, total (mg/dl)	1.00 (0.99–1.02)	0.6		
Triglyceride (mg/dl)	1.00 (0.99–1.01)	0.9		
HDL (mg/dl)	0.98 (0.94–1.03)	0.4		
LDL (mg/dl)	1.01 (0.99–1.04)	0.2		
Ferritin (ug/l)	1.00 (0.99–1.00)	0.3		
Homocysteine (umole/l)	1.07 (0.98–1.16)	0.1		
hsCRP (mg/L)	1.02 (1.00–1.04)	0.1		
Medications (vs. without)				
Statin/ezetimibe	1.92 (0.67–5.48)	0.2		
Aspirin	4.39 (1.38–13.99)	0.01	–	–
Clopidogrel	3.40 (1.19–9.70)	0.02	–	–
Cilostazol	1.37 (0.18–10.46)	0.8		
Unfractionated heparin	0.59 (0.21–1.69)	0.3		
LMWH	1.89 (0.65–5.43)	0.2		
ACEI/ARB	0.87 (0.35–2.16)	0.8		
Beta blocker	1.02 (0.39–2.62)	0.9		
CCB	0.77 (0.33–1.82)	0.6		
Comorbidity (vs. without)				
DM	4.08 (0.91–18.22)	0.07	5.60 (1.15–27.53)	0.03
Hypertension	4.22 (0.53–33.91)	0.4		
CHD	5.70 (1.77–18.17)	0.003	4.27 (1.29–14.09)	0.02
CVD	2.18 (0.49–9.77)	0.3		
PAD	0.46 (0.06–3.86)	0.5		

vWF = von Willebrand factor, HbA1c = hemoglobin A1c, HDL = high-density lipoprotein cholesterol, LDL = low-density lipoprotein cholesterol, hsCRP = high sensitivity C-reactive protein, LMWH = low molecular weight heparin, ACEI/ARB = angiotensin-converting enzyme inhibitor/angiotensin II receptor blocker, CCB = calcium-channel blocker, DM = diabetes mellitus, CHD = coronary heart disease, CVD = cerebral vascular disease, PAD = peripheral artery disease.

^a^Variables with a *p* value < 0.1 in univariate analysis were selected into the multivariate analysis by using the method of “forward stepwise selection” including ADAMTS13 activity, CHD, DM, current uses of clopidogrel and aspirin.

**Figure 2:**
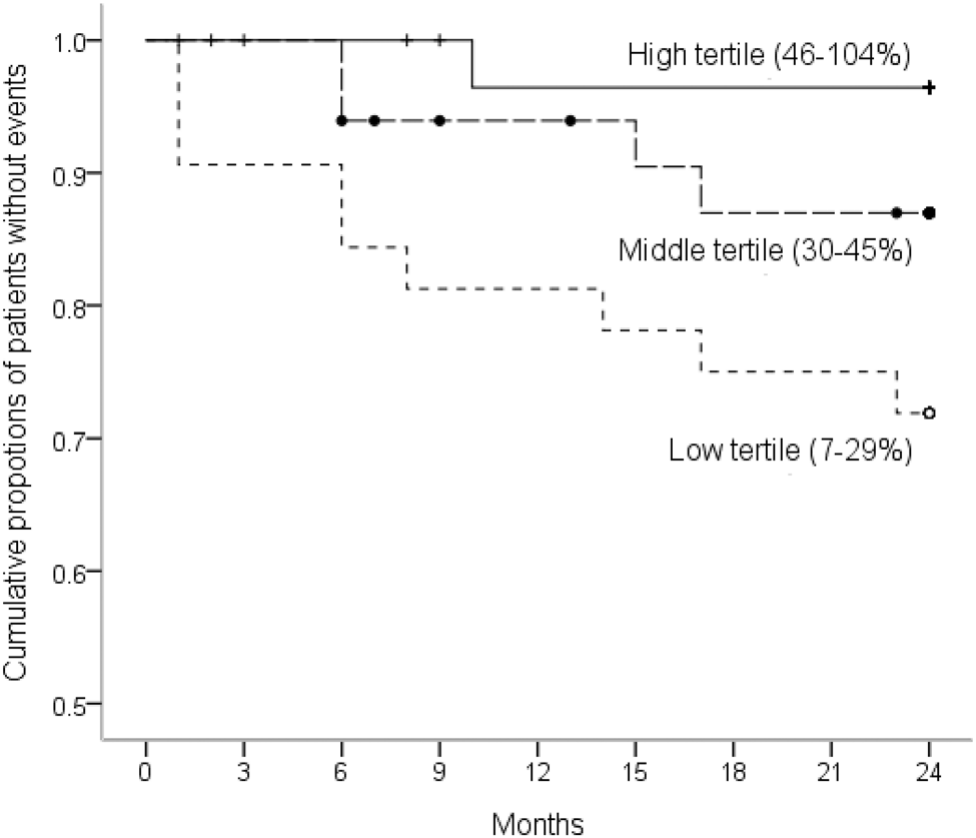
Cumulative proportions of patients without new cardiovascular events in 2-year follow-up period according to the tertile groups of ADAMTS13 activity, evaluated using a Kaplan–Meier survival curve, log rank test: *p* = 0.02.

While analyzed ADAMTS13 activity within tertile groups by multivariate Cox proportional hazards model, patients in the low tertile group (ADAMTS13 activity, range 7–29%) have a hazard ratio of 14.56 (95% CI 1.81–116.91, *p* = 0.01) to develop new cardiovascular events after adjusting comorbid DM and CHD (Table 3). In fact, patients in the low tertile suffered 5 CHD, 2 CVD, 1 PAD and 1 cardiac death; while, in the middle tertile developed 4 CHD and in the high tertile developed only 1 CVD. The Kaplan–Meier survival curves of cumulative proportions of event-free patients in the three groups were shown in Fig. 2.

**Table 3:** A multivariate Cox proportional hazards model for the occurrence of new cardiovascular events in the 2-year follow-up period according to the ADAMTS13 tertile groups in 98 chronic hemodialysis patients.

Variables^a^	Hazard ratio	95% Confidence interval	*p*
ADAMTS13 tertiles (range of ADAMTS13 activity)			
High tertile (46–104%)	1 (reference)		
Middle tertile (30–45%)	5.71	0.64–51.31	0.1
Low tertile (7–29%)	14.56	1.81–116.91	0.01
CHD (vs. without)	4.09	1.24–13.53	0.02
DM (vs. without)	4.59	0.97–21.83	0.06

CHD = coronary heart disease, DM = diabetes mellitus.

^a^Variables with a *p* value < 0.1 in univariate analysis at Table 3 were selected into this multivariate analysis using the method of “forward stepwise selection” including ADAMTS13 tertiles, CHD, DM, current uses of clopidogrel and aspirin.

## Discussion

In this study, our HD patients had significantly lower ADAMTS13 activity with a mean value of 41.0 ± 22.8%, far less than 102.3 ± 17.7% for healthy controls. This picture we observed was also shown by Rios et al. and Elzorkany et al.^17,18^. According to the study of Rios et al., the mean ADAMTS13 antigen level in 195 HD patients was 279 ng/ml, far less than 578 ng/ml in 80 healthy controls.

The causes why HD patients have a marked decrease of ADAMTS13 are not clear yet. ADAMTS13 synthesis was found initially in the liver, in platelet, and in endothelial cells of aorta and umbilical vein^19–23^. Subsequently, the expression and secretion of ADAMTS13 were also detected in human kidney, including podocytes, tubular epithelial cells, and glomerular endothelial cells^24–26^, which implicated a potential role of renal synthesis. In agreement with this hypothesis, Shen et al. found that chronic kidney disease (CKD) patients had a significantly lower ADAMTS13 activity than that of normal controls, no matter the etiology it was^27^. In addition, a significantly positive correlation between ADAMTS13 antigen levels and the estimated glomerular filtration rate was demonstrated by Taniguchi et al. in 86 patients with diabetic nephropathy. They also discovered that reduced ADAMTS13 levels are found only in patients with impaired renal function, not in whom with albuminuria only^28^. Overall, these findings suggested that ADAMTS13 deficiency in either CKD or HD patients might at least partially relate to their decreased renal synthesis.

In non-CKD patients, the association between low ADAMTS13 levels and enhanced MI risk had been demonstrated in a meta-analysis enrolled 5 studies with individual data on 1501 cases^29^. By using genetic variants to predict ADAMTS13 activity, it also confirmed this association^30^. Furthermore, reported by Sonneveld et al. in their large-scale prospective studies, a mild decreased ADAMTS13 activity to about 70% was found to be an independent risk factor for both ischemic stroke and CHD^13,14^. Compared to the above-mentioned studies, our HD patients had a much lower ADAMTS13 activity about 41%. It is reasonably to hypothesize this low activity can correlate to a high risk of cardiovascular diseases. Although our initial cross-sectional analyses failed to find the correlation between ADAMTS13 with CHD, CVD, or PVD, we demonstrated it in the prospective analyses that ADAMTS13 deficiency is an independent risk factor for the development of new cardiovascular events. The patients in the low tertile group, whose ADAMTS13 activity less than 30%, carry a significantly higher risk.

Even though the deficiency of ADAMTS13 might induce hypercoagulability and result in cardiovascular diseases in HD patients, the other mechanisms are remained to be clarified. Recently, ADAMTS13 deficiency has been reported in cases of severe sepsis, disseminated intravascular coagulation, complicated malarial infection, and systemic inflammation superimposed on advanced cirrhosis^31,32^. A prospective study on 72 septic shock patients showed that low ADAMTS13 activity correlated well to high interleukin-6 levels^33^. In a general health examination of 432 Japanese, low ADAMTS13 was also found correlated well to high CRP levels^34^. All above evidence suggested a pro-inflammation state of ADAMTS13 deficiency. Therefore, we supposed that the combination of pro-thrombotic and pro-inflammatory effects of ADAMT13 deficiency might further induce cardiovascular events in HD patients.

As we know, malnutrition, uremic toxins, and HD therapy itself could induce chronic micro-inflammation which might exaggerate the development of cardiovascular events in dialysis patients. However, no such correlation between cardiovascular events and serum albumin, lipids, hsCRP, homocysteine or HD vintage was observed in our study.

Surprisingly, our data showed that ADAMTS13 activity was positively correlated with HbA1c, fasting glucose, triglyceride levels, and comorbid DM and negatively with HDL level. In a prospective population-based study included 5176 participants, de Vries et al. demonstrated that ADAMTS13 activity could not only positively associated with fasting glucose level but also regarded as an independent risk factor for incident prediabetes and type 2 diabetes^35^. Crawley et al. also showed, in their case control study, high ADAMTS13 levels, but not low, were positively correlated with serum triglycerides, the presence of DM and negatively with HDL^10^. According to these 2 studies and ours, the associations between ADAMTS13 and hyperglycemia, hypertriglyceridemia and low HDL seem to be consistent in both non-CKD and HD patients. These findings suggest a potential correlation between ADAMTS13 and metabolic syndrome. In endothelial cells of human umbilical vein, ADAMTS13 could induce angiogenesis via an up-regulation of vascular endothelial growth factor (VEGF)^36^. And, VEGF had been suggested to have a major role in metabolic syndrome^37^. In short, high ADAMTS13 correlates to the components of metabolic syndrome, but low ADAMTS13 carries a significant cardiovascular risk. All the findings by now still cannot fully explain the causal relationships between ADAMTS13 and cardiovascular events. However, a recent animal study gave a ray of hope in this issue. Using recombinant human ADAMTS13, the investigators demonstrated an improvement of myocardial remodeling and functionality in mice after pressure overload injury^38^.

There were some limitations in our study. First, not all the risk factors for cardiovascular diseases were included in our study, such as smoking, lifestyle, or family history. Second, the data were collected in a single center and patient numbers were limited. Third, we did not follow up the healthy subjects for their cardiovascular events, therefore, we cannot make sure if there was a difference between HD patients and healthy subjects in terms of the occurrence of new cardiovascular events. Fourth, we focused on patients who undergoing HD and using native arteriovenous shunt as vascular access only, for the implanted artificial grafts or catheters might have shear stress and interfere with the ADAMTS13 function^39^. Therefore, our results cannot refer to all dialysis patients including those with peritoneal dialysis. Here, we encourage large scale studies to re-examine our results.

## Conclusions

Our chronic HD patients had a significantly lower ADAMTS13 activity than that in normal subjects. After adjusting multiple risk factors, patients in the low tertile (ADAMTS13 activity less than 30%) carried an independent risk for incident cardiovascular events during 2 years of follow-up. We suggest ADAMTS13 deficiency could be a novel risk factor for cardiovascular diseases in chronic HD patients. However, the correlations between ADAMTS13 and the components of metabolic syndrome were not only observed in non-CKD population but also in our HD patients. The explanations for this phenomenon are indebted to be explored.

## Methods

### Patients

All chronic HD patients in the dialysis center of E-DA Hospital were screened in Dec, 2015. Those who aged between 20 and 85, using native arteriovenous shunt for HD, and receiving HD thrice weekly for more than 3 months were included. Meanwhile, patients who using grafts or catheters as access for HD therapy, taking oral anticoagulants or contraceptives, having at least twofold higher than the upper limit of alanine aminotransferase level, a platelet count less than 80 × 10^3^/ml, or clinical evidences of infectious diseases within 1 month before entry, ever received solid organ transplantation, diagnosed to have thrombotic microangiopathies (such as thrombotic thrombocytopenic purpura and hemolytic uremic syndrome), liver cirrhosis, autoimmune diseases, malignancy for within 5 years, or uncured chronic inflammatory disease (such as tuberculosis and granulomatous diseases) were excluded. We also enrolled 100 healthy subjects as the control group. This study was approved and confirmed by the Ethics Committee of E-DA hospital (EMRP-104-084) that all experiments were performed in accordance with relevant guidelines and regulations. Informed consents were obtained from all participants. The research protocol did not interfere with any medical prescriptions or managements.

### Methods

The prescriptions for HD therapy such as blood and dialysate flow, non-reused dialyzer, heparin dosage during HD session, phosphate binders and vitamin D3 were decided by the Nephrologists in charge. Medical histories and clinical variables such as age, gender, HD vintage, underlying causes, comorbid diseases and current medications prescribed (including statin/ezetimibe, aspirin, clopidogrel, cilostazol, angiotensin-converting enzyme inhibitor, angiotensin II receptor blocker, beta blocker, and calcium-channel blocker) were obtained.

The comorbid diseases were defined as below. Diabetes mellitus (DM) was defined by history or currently using any anti-hyperglycemic agents. Hypertension was defined by history or currently taking any anti-hypertensive agents. CHD was defined by having undergone a coronary revascularization, coronary artery bypass graft, or having been diagnosed with angina pectoris or MI. Cerebral vascular disease (CVD) was defined as having undergone a carotid endarterectomy or having been diagnosed with carotid stenosis, transient ischemic attack or ischemic stroke. Peripheral artery disease (PAD) was defined as having peripheral arterial bypass surgery, amputation of digits or extremities secondary to vascular diseases or having been diagnosed with peripheral arterial occlusive disease.

A fasting, mid-week predialysis blood sample was collected in Jan, 2016 for measurement of complete blood cell count, biochemistry data, lipid profile, HbA1c, homocysteine, ferritin, high sensitivity C-reactive protein (hsCRP), ADMATS13 activity, vWf antigen level and activity. The 100 age- and sex-matched healthy controls also received a fasting blood measurement but only for ADAMTS13 activity.

Then, all the HD patients were followed up to 2 years after the collection of baseline data. The endpoints were the occurrence of any new cardiovascular events including CHD, CVD, PAD, and cardiac death.

### Assays

The biochemistry variables were measured by using commercial kits Architect C16000 (Abbott Laboratories, Lake Forest, IL, USA). Homocysteine, ferritin and hsCRP were determined through chemical luminescence and immunoturbidimetric methods by Abbott Architect Automatic analyzer (Abbott Laboratories, Lake Forest, IL, USA).

ADAMTS13 activity was measured by LIFECODES ATS-13 Activity Assay kit (Immucor^®^ GTI Diagnostics, Waukesha, WI, USA). Based on fluorescence resonance energy transfer (FRET) technology, ATS-13 activity assay is for the quantitative measurement of ADAMTS13 protease activity. A synthetic fragment of the vWf protein is used as the substrate. vWF antigen and activity (vWF ristocetin cofactor) were measured by immunoturbidimetric assay kits (Siemens, Marburg, Germany) in Sysmex CS‐2000i analyzer (Sysmex Corporation, Kobe, Japan).

### Statistical analyses

Student’s t-test or Chi-square test was performed to compare the data between HD patients and healthy subjects. The associations between ADAMTS13 activity and measured variables were evaluated by using the Pearson and Spearman's correlation tests with correlation coefficient r and r_s_, respectively. The data among the tertile groups were compared by using one-way ANOVA, Chi-square test or Kruskal–Wallis H test. After a follow-up period of 2 years, Cox proportional hazards regression analyses were used to evaluate the risk factors for new cardiovascular events. Patients were censored when deceased due to non-cardiovascular diseases, received transplantation, or were transferred to other hospitals. Variables with a p value less than 0.1 in univariate analysis were all tested using multivariate analysis. The Kaplan–Meier analysis with the log-rank test was then used to show the event-free curves of tertile groups. The general data were presented as the means ± standard deviations. Except in univariate analysis of Cox regression analysis, the statistical significance was set at a probability level of less than 0.05. The software SPSS 19.0 for Windows (SPSS Inc., Chicago, IL, USA) was used for statistical analysis.

## Data Availability

The datasets generated during and/or analyzed during the current study are available from the corresponding author on reasonable request.
